# Cryosurgery as a Therapeutic Alternative in Extensive Lymphangioma of the Dorsal Tongue: A Case Report

**DOI:** 10.7759/cureus.91351

**Published:** 2025-08-31

**Authors:** Amara Hazel Solorio-Rivera, Julio César Aguilar-Pérez, Jabes Israel Basaldua-Ibarra, María Fernanda Limón-Limón, María Del Consuelo García-Silva

**Affiliations:** 1 Dermatology, Instituto Dermatológico de Jalisco “Dr. José Barba Rubio”, Zapopan, MEX; 2 Dermatologic Surgery, Instituto Dermatológico de Jalisco “Dr. José Barba Rubio", Zapopan, MEX; 3 Dermatology, Instituto Dermatológico de Jalisco "Dr. José Barba Rubio", Zapopan, MEX

**Keywords:** cryosurgery, lymphangioma, lymphatic malformation, oral surgical procedures, tongue mass

## Abstract

Lymphangiomas are benign malformations of the lymphatic system that most frequently occur in the head and neck. The dorsal surface of the tongue is the most commonly affected site in the oral cavity. Its management represents a therapeutic challenge due to the infiltrative nature and recurrence potential. Although surgical excision is the standard treatment, taking into account the associated risks, other less invasive options have been suggested, such as cryosurgery. We report the case of a 21-year-old woman with a dorsal tongue lymphangioma presenting since birth, treated with three sessions of cryosurgery, achieving significant volume reduction without compromising tongue functionality. Cryosurgery offers an effective and safe therapeutic alternative compared to other treatment options, with favorable functional and aesthetic outcomes.

## Introduction

Lymphangiomas are benign malformations of the lymphatic system resulting from abnormal sequestration of lymphatic tissue, leading to the proliferation of dilated channels. This explains their infiltrative growth pattern and high tendency to recur, making management particularly challenging [1]. Approximately 75% occur in the head and neck region, and within the oral cavity, the dorsal surface of the tongue is the most commonly affected site, in around 50% of the cases [2,3]. Lymphangiomas are usually detected at birth or in early childhood, and by two years of age, 90% have already developed [4]. On the tongue, they typically present as nodular or diffuse lesions with superficial vesicles containing serosanguinous fluid, which can lead to macroglossia, impaired swallowing, and, in advanced stages, airway obstruction [5]. The diagnosis is primarily clinical, supported by imaging, and confirmed by histopathology [6]. Multiple treatment options are available, including surgery, sclerotherapy, laser therapy, intralesional corticosteroids, bleomycin, and cryosurgery. Although surgical excision has traditionally been the standard approach, it can result in significant functional and aesthetic complications [7], whereas cryosurgery represents a safe and effective therapeutic alternative, offering lower morbidity and better functional and aesthetic outcomes compared with surgical resection [8,9]. While several treatment modalities such as surgery, sclerotherapy, and laser have been described, cryosurgery provides a minimally invasive alternative with fewer complications. However, evidence on cryosurgery as monotherapy is limited, particularly in extensive lingual lymphangiomas in adults, highlighting the novelty and clinical value of this report. 

## Case presentation

**Figure 1:**
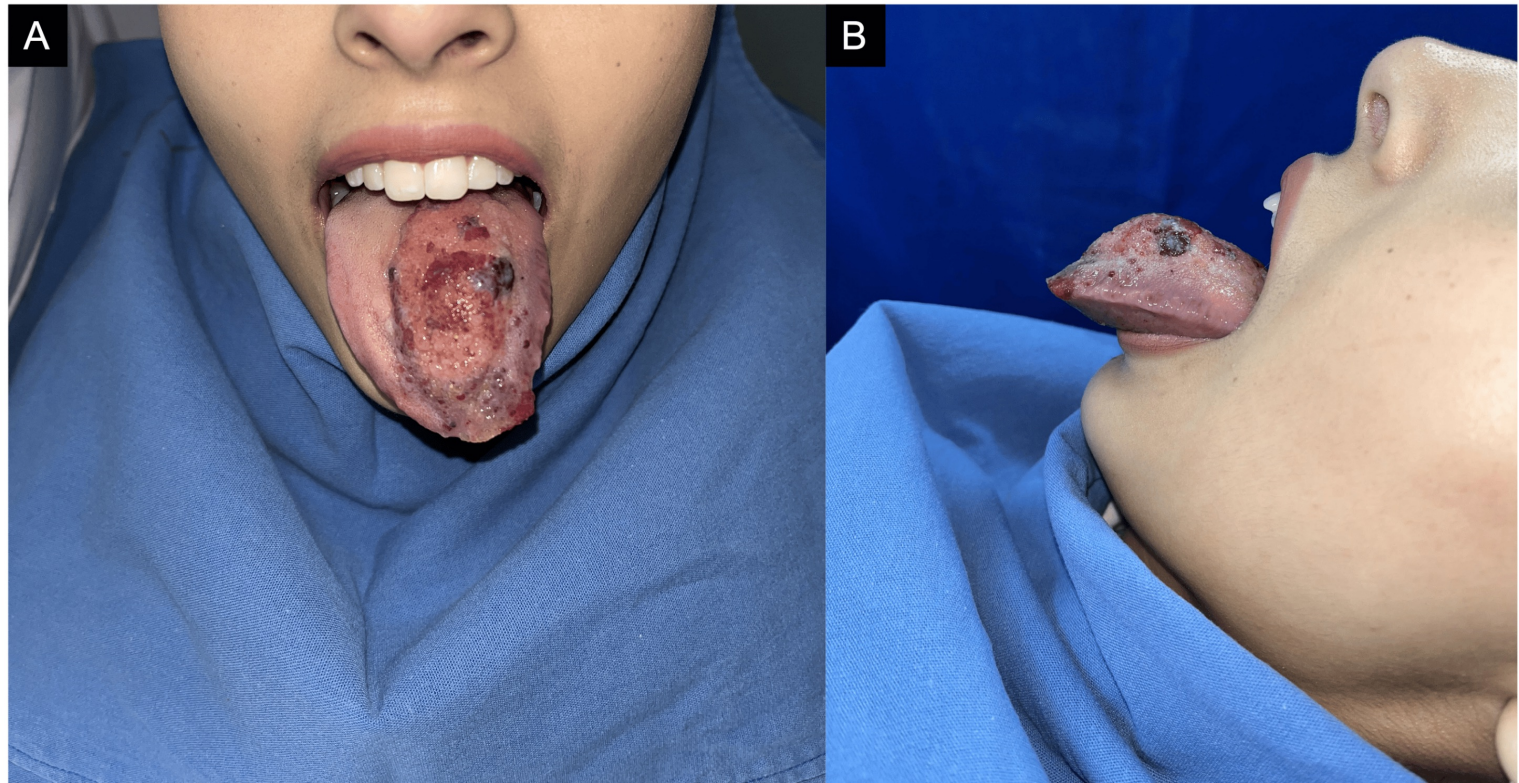
(A) Multilobulated mass measuring 5×4×2.5 cm, located on the left mid-third of the dorsal tongue. (B) Lateral view of the lesion showing its multilobulated, exophytic appearance.

A 21-year-old woman with no relevant medical history presented for evaluation of a congenital lesion on the dorsal tongue present since birth, which she mentioned had shown progressive growth over the past few years. The patient had not received any prior treatment for the lesion. The lesion caused increasing difficulty in swallowing and speech articulation. Physical examination revealed a 5×4×2.5 cm exophytic, multilobulated, vascular-appearing mass with yellowish and reddish vesicles on its surface and ill-defined borders, localized on the left hemidorsum of the tongue (Figure 1).

**Figure 2:**
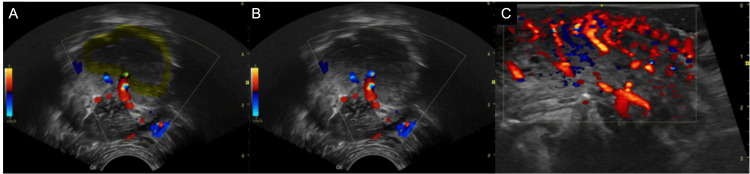
Color Doppler ultrasound of the dorsal tongue. (A,B) Multiple prominent, tortuous vascular channels within the tongue musculature, showing both arterial and venous flow with arteriovenous shunts. (C) Longitudinal view demonstrating extensive vascularity involving the mid and anterior thirds of the tongue.

**Figure 3:**
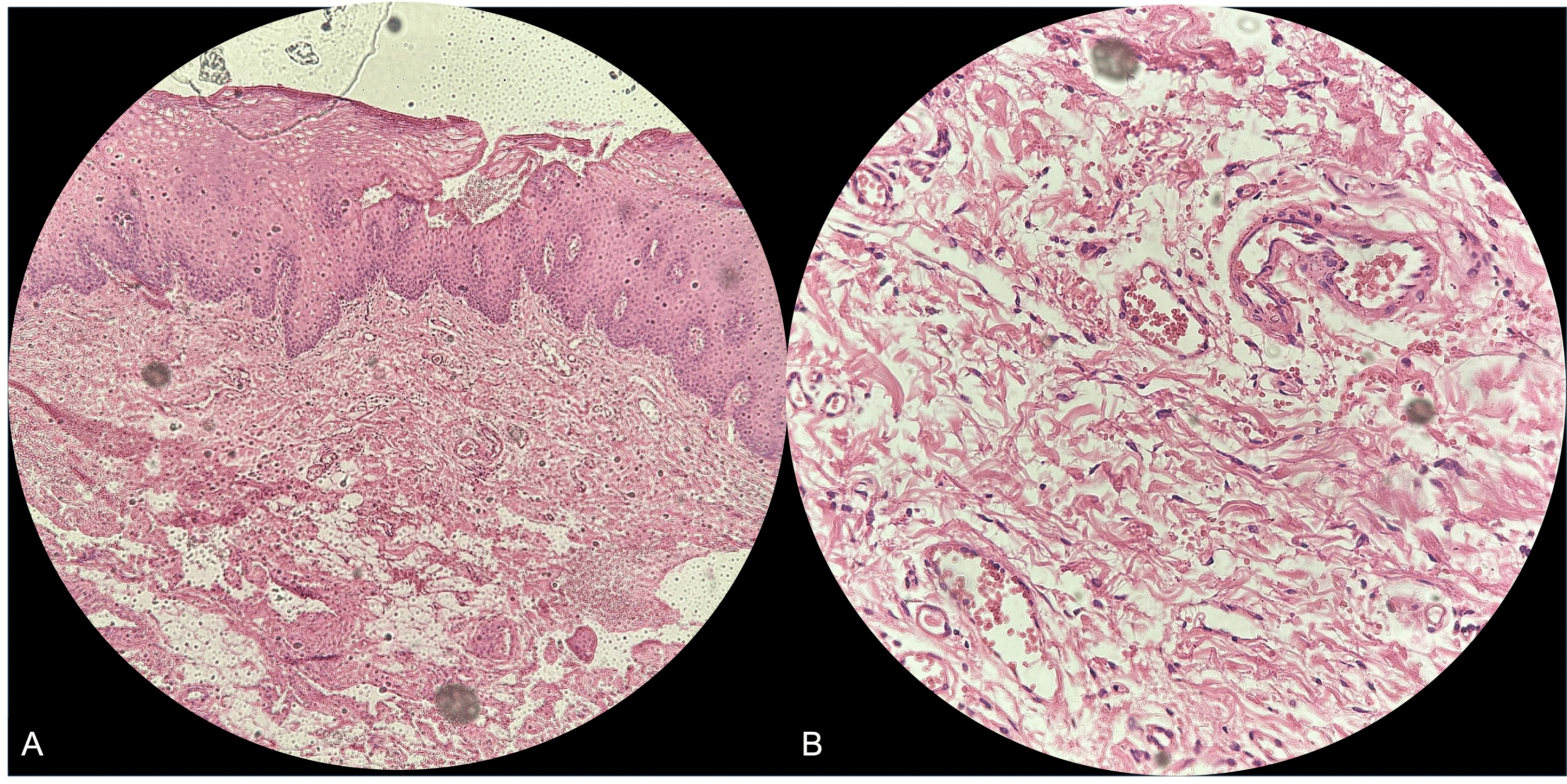
Histopathological features of the lesion. (A) Low-power view showing numerous dilated lymphatic channels within the lamina propria beneath stratified squamous epithelium. (B) Higher magnification shows a flattened endothelial lining and clear proteinaceous fluid within vascular spaces, consistent with lymphangioma (H&E, ×10; inset ×40).

Doppler ultrasound showed involvement of multiple intrinsic tongue muscles and demonstrated arterial and venous flow with arteriovenous shunts, supplied by direct branches of the left lingual artery. The lesion measured approximately 4.6 cm in length, 2.3 cm in width, and 3.1 cm in thickness (Figure 2), and histopathological examination revealed dilation of lymphatic channels throughout the upper portion of connective tissue, along with a chronic inflammatory infiltrate composed predominantly of lymphocytes and plasma cells in the stroma (Figure 3).

**Figure 4:**
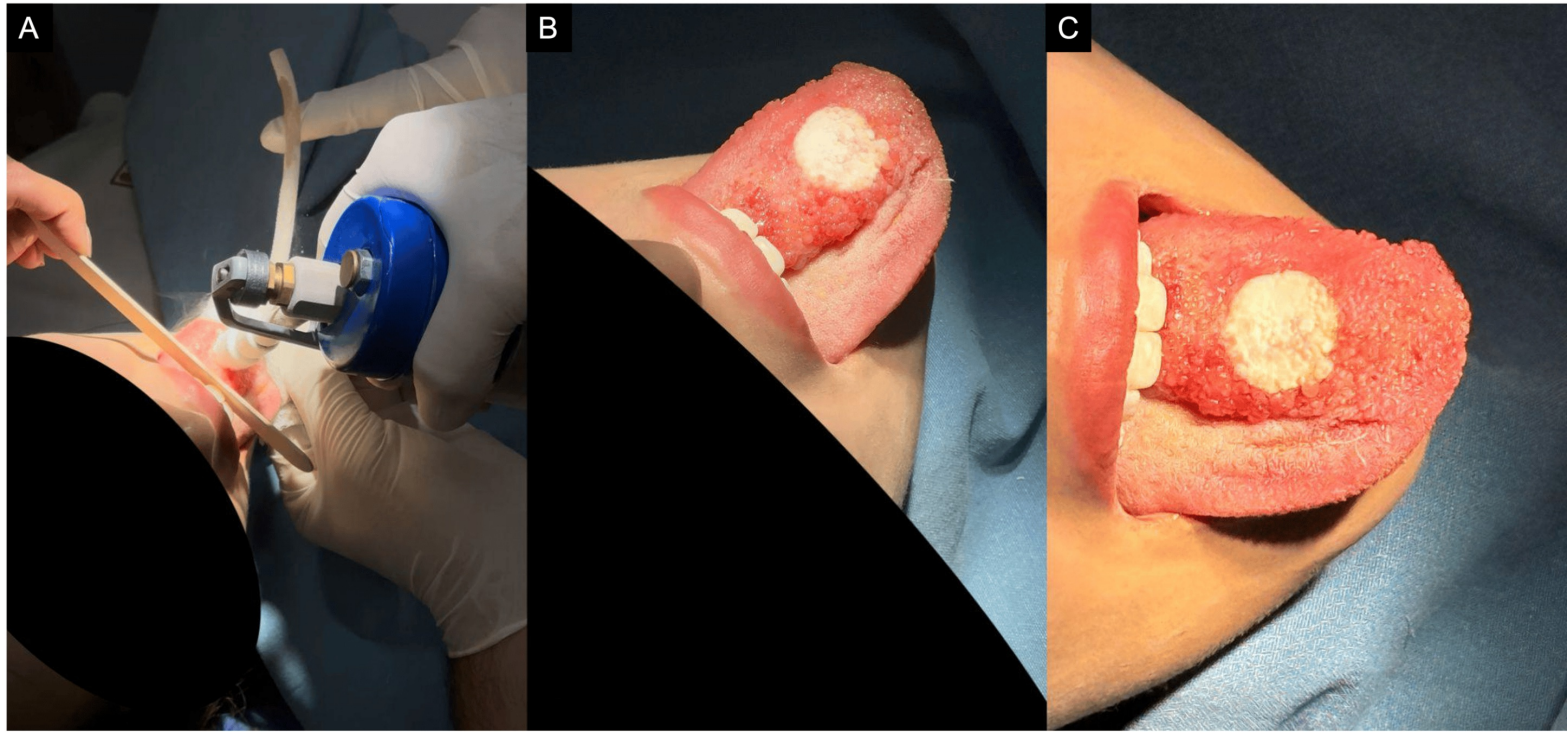
(A) Initial freezing phase using the closed cryosurgical technique. (B) Solid ice-ball formation extending to the lesion margins. (C) Passive thawing phase following cryoapplication.

The patient underwent three sessions of cryosurgery, administered at six-week intervals, performed under regional anesthesia through mandibular nerve block. A closed cryosurgical technique was performed, achieving a visible ice-ball formation with a 0.2 cm margin beyond the cryoprobe tip, followed by passive thawing. Two freeze-thaw cycles were applied to the central bulky portion and a single cycle to the lateral and apical areas (Figure 4).

**Figure 5:**
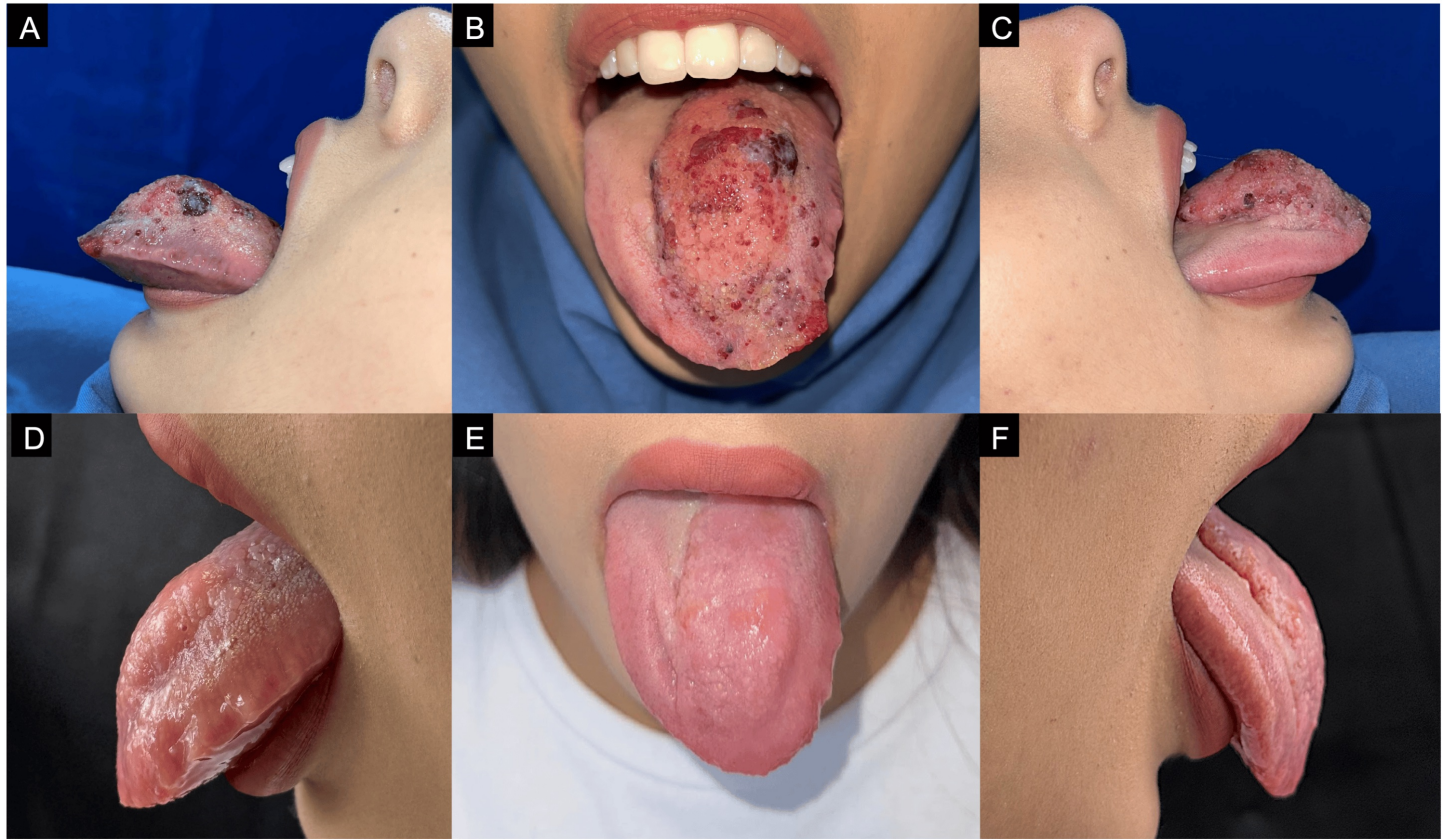
(A-C) Anterior and lateral views of the lesion before treatment, showing initial lesion volume. (D-F) Clinical appearance two months after the third cryosurgery session, demonstrating a significant reduction in lesion size compared with baseline.

At follow-up, two months after the final session, clinical examination showed a marked reduction in lesion volume, as documented in follow-up photographs (Figure 5). The patient reported complete resolution of swallowing difficulties and noted a significant improvement in her social and emotional quality of life after treatment. As part of the natural healing process after cryosurgery, transient edema was observed during the first 10 days; however, no necrosis or nerve injury occurred.

## Discussion

Most oral cavity lymphangiomas manifest during childhood and are typically located on the dorsal surface of the tongue [1,3]. Clinically, they may present as exophytic vascular lesions with superficial vesicles and progressive enlargement [4,5]. When lesions are extensive, they can impair basic functions such as mastication, speech, and swallowing [4,5], as in the case of our patient. While surgical excision has traditionally been the treatment of choice for lymphangiomas, in cases of extensive tongue involvement, it may result in significant complications such as dysarthria, dysphagia, and, paradoxically, an increased risk of recurrence [7]. Therefore, we opted for cryosurgery as a more conservative technique with lower morbidity. The effectiveness of cryosurgery in lymphatic malformations is thought to result from endothelial cell destruction, vascular thrombosis, and subsequent fibrosis of the abnormal lymphatic channels, leading to progressive shrinkage of the lesion [1].

A structured literature search was performed in PubMed and Google Scholar (2000-2024) using the terms "lymphangioma," "tongue," and "cryosurgery." Only a limited number of reports describing lingual lymphangiomas treated with various modalities were identified. These cases are summarized in Table 1.

**Table 1:** Reported cases of tongue lymphangioma treated with different modalities.

Author (year)	Patient age/sex	Lesion site	Treatment modality	Outcome	Follow-up
Bhayya et al. (2015) [3]	6 y/F	Tongue (oral)	Surgical excision	Good functional recovery	Not specified
Goswami et al. (2011) [5]	12 y/M	Tongue	Surgical excision	Resolution	Not specified
Hwang et al. (2017) [9]	24 y/M	Tongue	Intralesional steroid + bleomycin + bevacizumab	Reduction in size, improved function	6 months
Júnior et al. (2023) [8]	8 y/M	Dorsal tongue	Cryosurgery + diode laser	Good functional results, no recurrence	8 years
Present case (2025)	21 y/F	Dorsal tongue	Cryosurgery (3 sessions)	Marked reduction in lesion size, improved swallowing and quality of life, no complications	2 months

Nunes Júnior et al. [8] reported a case of an extensive dorsal tongue lymphangioma treated with cryosurgery combined with 655 nm laser, attaining good functional results and no evidence of recurrence in the long term. Our patient underwent three sessions of cryosurgery as monotherapy, achieving substantial clinical improvement with a marked reduction in lesion size, preservation of tongue functionality, and no recurrence at a two-month follow-up.

Compared to sclerotherapy and laser therapy, cryosurgery provides similar rates of lesion control, but with fewer functional complications and shorter healing times. While sclerotherapy is often effective for smaller or localized lesions, it may require multiple sessions and carries a risk of local tissue reaction. Laser therapy can achieve precise ablation but may result in scarring or recurrence. In contrast, cryosurgery combines effectiveness with preservation of function and good cosmetic outcomes. Potential complications of cryosurgery include edema, tissue necrosis, delayed healing, and, in rare cases, nerve injury [2]. In our case, only transient edema was observed during the first 10 days, while no necrosis or nerve damage occurred.

No other reports were found describing lingual lymphangiomas treated exclusively with cryosurgery; this technique represents a safe and effective alternative for anatomically challenging lesions with high surgical risk [1,7,8]. Other treatment options include combination therapies such as sclerotherapy or intralesional agents like corticosteroids and bleomycin, which have shown effectiveness in selected cases with smaller lesions [9].

This case supports the use of cryosurgery as a stand-alone therapeutic modality for extensive dorsal tongue lymphangiomas. It is a safe and effective technique associated with minimal complications, preservation of lingual function, and excellent cosmetic outcomes. Due to the limited evidence published on this therapeutic option, further clinical studies are needed to evaluate its long-term efficacy. In particular, longer follow-up periods are essential to confirm the durability of clinical improvement and to assess recurrence risk.

## Conclusions

Cryosurgery represents an effective and minimally invasive therapeutic option for the treatment of lingual lymphangiomas. Treatment should be individualized based on lesion type, location, and extension. Current clinical experience and available evidence support its use as a safe alternative with favorable functional and aesthetic outcomes. However, the main limitations of this report are its single-case nature and the short follow-up period, which do not allow definitive conclusions regarding recurrence. Further studies with larger patient series and longer follow-up are needed to validate these findings.
